# From vision–language models to structure-aware visual reasoning: foundations, representations, and consistency checking

**DOI:** 10.3389/frobt.2026.1781874

**Published:** 2026-06-24

**Authors:** Xiaoyan Dai

**Affiliations:** KYOCERA Corporation, Yokohama, Japan

**Keywords:** embodied AI, multimodal perception, neuro-symbolic integration, safety-critical systems, structure-aware reasoning, structured inference, temporal reasoning, uncertainty propagation

## 1 Introduction

Vision-Language Models (VLMs) have become a central paradigm in multimodal artificial intelligence. Large-scale image-text pretraining has enabled strong performance in image captioning, visual question answering, visual instruction following, and increasingly complex multimodal reasoning. These advances have encouraged the use of VLMs in robotics and embodied AI, where perceptual input must be translated into decisions and actions in the physical world (Radford et al., 2021; Li J. et al., 2023).

A balanced assessment must acknowledge that recent VLMs are much stronger than earlier models. Systems such as GPT-4V and Gemini 1.5 show impressive multimodal understanding, long-context reasoning, and useful spatial or relational reasoning under many controlled conditions. Open-source models such as LLaVA have made visual instruction following widely accessible. In robotics, PaLM-E and RT-2 demonstrate that vision-language pretraining can support semantic generalization and simple instruction-conditioned actions. These systems are important counterexamples to any claim that VLMs lack useful reasoning ability (OpenAI, 2023; Gemini Team, 2024; Liu et al., 2023; Driess et al., 2023; Brohan et al., 2023).

Nevertheless, robotics and safety-critical systems require more than high average benchmark accuracy. They require reliable behavior under distribution shift, occlusion, clutter, temporal change, partial observability, and safety constraints. A model may answer a relational question correctly in one prompt yet fail under a logically equivalent prompt, an edited scene, or a multi-step action sequence. When perception, reasoning, and decision-making remain compressed into a monolithic latent representation, failures are difficult to localize, audit, or constrain (Amodei et al., 2016; Bender et al., 2021).

This Opinion therefore narrows its criticism to end-to-end VLMs used as direct decision makers without explicit intermediate state, uncertainty estimates, or verification mechanisms. The argument is not that VLMs should be abandoned. Rather, VLMs should be used as powerful perceptual and semantic front ends whose outputs are made explicit, checked, and integrated with structured reasoning before they drive high-stakes decisions or robot actions. We call this complementary paradigm Structure-Aware Visual Reasoning (SAVR). SAVR treats VLM outputs as hypotheses rather than final facts and organizes them into explicit structures over entities, attributes, relations, temporal state, constraints, and uncertainty (Doshi-Velez and Kim, 2017; Marcus, 2020).

## 2 Limitations of end-to-end vision–language models

End-to-end VLMs can align visual and linguistic information effectively, but their implicit reasoning creates recurring limitations. They may hallucinate objects, misbind attributes, confuse relations, or produce inconsistent answers to equivalent queries. They also lack explicit mechanisms for enforcing physical or logical constraints, such as object permanence, support, containment, conservation, or action preconditions. In embodied settings, these weaknesses become safety concerns because a plausible language output can lead to an unsafe action.

### 2.1 Recent advances observed in VLMs

A major counterargument is that frontier VLMs already display spatial, relational, and even physical reasoning. This point is valid. Chain-of-thought prompting can improve reasoning by eliciting intermediate steps, and multimodal models sometimes benefit from similar prompting. GPT-4V and Gemini-class systems show strong visual understanding, while RT-2 shows that vision-language-action models can transfer web knowledge to robotic control by representing actions in a token-like format. These results show the power of end-to-end learning and large-scale pretraining.

The SAVR claim is therefore deployment-oriented, not a universal negative claim about VLMs. For robotics and other safety-critical systems, emergent capabilities are valuable but insufficient if the system cannot expose its state, represent uncertainty, enforce constraints, or explain why proposed action is safe. SAVR seeks to preserve VLM strengths while adding the interfaces needed for verification and control.

### 2.2 Concrete failure modes observed in VLMs

Concrete benchmark evidence motivates this concern. Object hallucination has long been measured in captioning with the CHAIR metric, and POPE evaluates hallucination in large VLMs by asking yes/no questions about object presence, including absent objects sampled in random, popular, or adversarial ways. These studies show that models can affirm objects that are not visually supported, especially when objects commonly co-occur with visible objects or appear frequently in instruction data (Rohrbach et al., 2018; Li Y. et al., 2023).

Relational and image-context errors also persist. HallusionBench evaluates entangled visual illusion and language hallucination in advanced VLMs, including GPT-4V, Gemini Pro Vision, Claude 3, and LLaVA-1.5. It includes cases where models must separate visual evidence from prior assumptions. Attribute binding remains another challenge: a model may recognize a red cup and a blue bowl but assign the wrong color to the wrong object. Benchmarks such as CLEVR and GQA illustrate why visual reasoning requires composition, relation tracking, and consistency across queries, not only isolated recognition (Guan et al., 2024; Fu et al., 2023; Yi et al., 2017; Hudson and Manning, 2019).

In robotics, such errors are not merely linguistic. A household robot asked to pick up the cup to the left of the plate must bind object identity, position, affordance, and temporal state. If it hallucinates a cup, misbinds a relation, or fails to notice that the cup has moved behind an obstacle, a plausible response may still produce an unsafe action.

## 3 Structure-aware visual reasoning

SAVR is a hybrid design philosophy rather than a single algorithm. It makes intermediate reasoning states inspectable and checkable while still using VLMs as high-capacity perceptual and semantic modules. Explicit structure provides an interface between learned perception and verifiable reasoning. It helps developers inspect state, localize errors, encode domain knowledge, apply constraints, and decide when the system should ask for more information rather than act.

In this Opinion, structure is defined operationally rather than as one mandatory data structure. At minimum, a SAVR system maintains a time-indexed relational state S_t = (E_t, A_t, R_t, C_t, U_t). E_t denotes entity hypotheses with persistent identifiers. A_t denotes typed attributes such as color, pose, material, state, or affordance. R_t denotes typed relations such as left-of, inside, supporting, touching, occluding, or causing. C_t denotes physical, logical, task, or safety constraints. U_t denotes uncertainty, provenance, or confidence associated with entities, attributes, relations, and constraints.

This state can be implemented as a scene graph, temporal scene graph, knowledge graph, factor graph, probabilistic relational model, or first-order logic predicate set. The key requirement is not the label of the representation but the ability to individualize entities, bind attributes to the correct entities, represent relations, track time, attach uncertainty, and check constraints. For example, instead of accepting the caption “a red cup is on the table” as a final decision, SAVR would represent entity e1 of type cup, color (e1) = red, on (e1, table1), confidence for each claim, visual provenance, and constraints such as stable support (e1, table1). If evidence conflicts across frames, the system can mark the claim as uncertain and trigger re-observation (Johnson et al., 2015; Battaglia et al., 2018; Kipf et al., 2018; De Raedt et al., 2020; Besold et al., 2021).

### 3.1 Temporal and embodied reasoning

Embodied AI requires temporal reasoning beyond static scene description. Objects can become occluded, move outside the field of view, change state, or be transformed by actions. A robot must reason about object permanence, causal effects, action preconditions, and state transitions. Opening a drawer changes visibility and accessibility; grasping a cup changes its support relation; pouring water changes the state of both the cup and the liquid.

SAVR extends entity-attribute-relation representations into temporal state models. Entities should persist across frames through tracking or re-identification. Relations can be time-indexed, such as inside (e1, drawer1, t). Actions can have preconditions and expected effects, such as open (drawer1) making visible (object_x) more likely. Such temporal structures allow a system to detect contradictions, including an object being simultaneously inside a closed drawer and visible on a table. Temporal coherence, object permanence, causal consistency, and action-effect prediction should therefore be core evaluation axes (Pearl and Mackenzie, 2018; Huang et al., 2022).

### 3.2 Perceptual uncertainty and graceful degradation

A legitimate concern is that SAVR may simply delegate the hard perception problem to VLMs. If a VLM hallucinates objects or misbinds attributes, why should its output be trusted as input to a structured reasoner? SAVR addresses this concern by treating VLM outputs not as reliable facts but as uncertain perceptual hypotheses that must be verified, updated, or rejected.

In practice, the structured state should store confidence, provenance, and alternatives. An entity may be represented as cup (e1, p = 0.72) or bowl (e1, p = 0.28), while inside (e1, box1) may remain uncertain until confirmed from another viewpoint or sensor. SAVR can combine VLM hypotheses with object detectors, segmentation models, depth sensors, trackers, or task-specific classifiers. When sources disagree, contradictions should trigger active perception or conservative fallback. If the system is uncertain whether a knife is on a table, a robot should re-observe, ask for confirmation, or choose a safe motion plan rather than act as if the fact were certain.

Uncertainty also changes how constraints are applied. Hard constraints are appropriate for high-confidence facts and safety-critical rules. In ambiguous cases, soft constraints can down-weight inconsistent hypotheses, prioritize information gathering, or block unsafe actions. This enables graceful degradation: the system can reduce autonomy, abstain, request human assistance, or execute only actions whose safety does not depend on uncertain claims.

## 4 Discussion

SAVR is best understood as a hybrid design philosophy rather than a single algorithm. It lies between fully end-to-end VLMs and purely symbolic systems. It preserves the flexibility of learned perception while introducing explicit state, constraints, and uncertainty handling where verification matters.

### 4.1 Related structured reasoning paradigms

SAVR lies between fully end-to-end VLMs and purely symbolic systems. It preserves the flexibility of learned perception while introducing explicit state, constraints, and uncertainty handling where verification matters. It is also related to several important paradigms.

Chain-of-thought prompting shows that exposing intermediate reasoning steps can improve complex reasoning, but textual traces do not guarantee visual grounding. A chain of thought may be fluent while relying on a hallucinated object or misbound attribute. SAVR differs by grounding intermediate steps in explicit perceptual hypotheses and constraints (Wei et al., 2022; Lake et al., 2017).

Visual programming approaches such as VisProg and ViperGPT are close relatives. They use language models to generate executable programs that call visual modules and compose intermediate results. Their success supports the broader claim that modular, inspectable reasoning can complement monolithic inference. SAVR extends this idea toward embodied systems by emphasizing persistent state, uncertainty, physical constraints, and safety (Gupta and Kembhavi, 2023; Suris et al., 2023).

LLaVA demonstrates the practical power of visual instruction tuning in open VLMs. SAVR can use such models as proposal generators, but the structured layer need not treat the VLM as the sole authority. RT-2 is especially relevant because it shows that vision-language-action models can transfer web knowledge to robotic control. This is a strong counterexample to any claim that end-to-end VLM robotics is unpromising. The narrower claim here is that RT-2-like systems still benefit from explicit state, uncertainty, and verification when actions have safety consequences.

### 4.2 Evaluation axes for SAVR

Traditional benchmarks emphasize answer accuracy, but accuracy alone is insufficient for structured embodied reasoning. SAVR-based systems should also be evaluated on entity grounding, attribute-binding accuracy, relation consistency, temporal coherence, constraint satisfaction, uncertainty calibration, failure detectability, safe abstention, and action-level safety. Concrete evaluations could include logically equivalent queries, counterfactual image edits, multi-frame object permanence tests, relation-swapping scenes, and action-precondition tests. A system should not only answer correctly but also maintain consistent state and know when evidence is insufficient.

The argument for SAVR is falsifiable. It would be weakened if future end-to-end VLM or vision-language-action systems consistently achieved reliable embodied performance under distribution shift, occlusion, clutter, temporal state changes, causal interventions, and adversarial relation tests while also providing calibrated uncertainty, safe abstention, and auditable internal states sufficient for certification or failure analysis. In that case, explicit structure might become less necessary as an external layer because similar capabilities would have been internalized and made accessible. Until such evidence exists, SAVR remains a practical strategy for connecting foundation-model strengths to reliability requirements.

### 4.3 Future directions and open challenges

Several challenges remain. First, learning structured representations from raw sensory data is difficult under occlusion, clutter, and domain shift. Second, reasoning over large temporal structures can be computationally expensive. Approximate inference, hierarchical representations, and event-driven updates may be needed to maintain tractability. Third, constraints should not all be hand-coded. Future systems may learn soft constraints and priors from data while preserving hard safety rules for critical cases.

A further challenge is integration with large foundation models. SAVR should not suppress the emergent capabilities of VLMs; rather, it should provide a structured interface through which those capabilities can be checked, combined, and safely acted upon. Designing this interface is likely to be a central research problem for trustworthy multimodal and embodied AI.

## 5 Conclusion

Foundation VLMs have substantially advanced multimodal perception and reasoning, and recent systems show genuine progress in spatial, relational, and embodied tasks. Nevertheless, robotics and safety-critical systems require more than plausible language outputs or high average benchmark scores. They require inspectable state, temporal coherence, uncertainty handling, constraint satisfaction, and safe behavior when perception is incomplete.

SAVR is proposed as a complementary paradigm that treats VLM outputs as uncertain perceptual hypotheses and organizes them into explicit, verifiable structures. By connecting the representational power of foundation models with structured reasoning, uncertainty propagation, and consistency checking, SAVR offers a practical path toward more reliable multimodal systems for robotics and embodied AI.
